# Nogo-A Neutralization Improves Graft Function in a Rat Model of Parkinson’s Disease

**DOI:** 10.3389/fncel.2016.00087

**Published:** 2016-04-05

**Authors:** Stefanie Seiler, Stefano Di Santo, Hans Rudolf Widmer

**Affiliations:** ^1^Department of Neurosurgery, Neurocenter and Regenerative Neuroscience Cluster, University Hospital BernSwitzerland; ^2^Graduate School for Cellular and Biomedical Sciences, University of BernBern, Switzerland

**Keywords:** Parkinson’s disease, Nogo-A, cell transplantation, dopaminergic neurons, behavior, rat

## Abstract

Transplantation of fetal human ventral mesencephalic (VM) dopaminergic neurons into the striatum is a promising strategy to compensate for the characteristic dopamine deficit observed in Parkinson’s disease (PD). This therapeutic approach, however, is currently limited by the high number of fetuses needed for transplantation and the poor survival and functional integration of grafted dopaminergic neurons into the host brain. Accumulating evidence indicates that contrasting inhibitory signals endowed in the central nervous system (CNS) might support neuronal regeneration. Hence, in the present study we aimed at improving survival and integration of grafted cells in the host brain by neutralizing Nogo-A, one of the most potent neurite growth inhibitors in the CNS. For that purpose, VM tissue cultures were transplanted into rats with a partial 6-hydroxydopamine (6-OHDA) lesion causing a hemi-PD model and concomitantly treated for 2 weeks with intra-ventricular infusion of neutralizing anti-Nogo-A antibodies. Motor behavior using the cylinder test was assessed prior to and after transplantation as functional outcome. At the end of the experimental period the number of dopaminergic fibers growing into the host brain, the number of surviving dopaminergic neurons in the grafts as well as graft size was examined. We found that anti-Nogo-A antibody infusion significantly improved the asymmetrical forelimb use observed after lesions as compared to controls. Importantly, a significantly three-fold higher dopaminergic fiber outgrowth from the transplants was detected in the Nogo-A antibody treated group as compared to controls. Furthermore, Nogo-A neutralization showed a tendency for increased survival of dopaminergic neurons (by two-fold) in the grafts. No significant differences were observed for graft volume and the number of dopaminergic neurons co-expressing G-protein-coupled inward rectifier potassium channel subunit two between groups. In sum, our findings support the view that neutralization of Nogo-A in the host brain may offer a novel and therapeutically meaningful intervention for cell transplantation approaches in PD.

## Introduction

Parkinson’s disease (PD) is a neurodegenerative disorder mainly characterized by the progressive loss of dopaminergic neurons in the substantia nigra pars compacta (SNc) with a subsequent loss of dopamine innervation in the striatum. To restore the imbalanced innervation in the striatum, cell replacement strategies have been evaluated as a long-term treatment for PD. Numerous clinical trials showed functional improvement after transplantation of fetal human ventral mesencephalic (VM) dopaminergic neurons into the striatum of PD patients (Lindvall et al., 1989, 1990; Kordower et al., 1995; Hauser et al., 1999) even without the support of additional pharmacological treatment (Kefalopoulou et al., 2014). It became clear, however, that before cell replacement strategies can be used in clinical practice, the criteria for patient selection (Freed et al., 2001; Barker et al., 2013) as well as the collection, the pre-treatment and the storage of donor tissue have to be optimized and standardized (Petit et al., 2014). Currently, a multicenter clinical trial called TRANSEURO is establishing a standardized protocol for transplantation approaches in PD (Moore et al., 2014). Two other major limitations associated with cell transplantation procedures in PD still include suboptimal survival and poor innervation of the host brain by grafted dopaminergic neurons. Several transplantation studies in animals revealed that most of the transplanted dopaminergic neurons die within the first week after transplantation and that many of the surviving dopaminergic neurons do not functionally integrate into the host brain (Emgard et al., 1999; Karlsson et al., 2000). In line with this notion, it has been shown that mainly the dopaminergic sub-population expressing the G-protein-coupled inward rectifier potassium channel subunit 2 (GIRK2) innervate the host brain after transplantation (Mendez et al., 2005; Thompson et al., 2005; Gaillard et al., 2009; Grealish et al., 2010).

Accumulating evidence points out that the tissue microenvironment plays a vital role in influencing the degree of engraftment of the transplant (Ourednik and Ourednik, 2005; Alsberg et al., 2006; Stefanova et al., 2009). Suppression of myelin associated proteins like Nogo-A, a major neurite growth inhibitor in the central nervous system (CNS) might therefore offer novel ways to improve functional recovery in various disease states. In line with this notion, several studies have demonstrated that neutralizing Nogo-A signaling with anti-Nogo-A antibodies promoted axonal sprouting as well as improved functional recovery in spinal cord injured rats and monkeys (Merkler et al., 2001; Freund et al., 2009). Moreover, Nogo-A antibodies increased neuronal remodeling and functional recovery after stroke in rodents (Cheatwood et al., 2008; Tsai et al., 2011). Hence, we hypothesized that this strategy may be considered a valuable approach in the context of cell transplantation in PD. Nogo-A, is widely distributed in the CNS and it is expressed in dopaminergic neurons of the rodent fetus as well as in dopaminergic neurons of the adult rodent SNc (Seiler et al., 2013; Kurowska et al., 2014; Schawkat et al., 2015). Notably, knock-out animals for leucine rich repeat neuronal protein 1 (LINGO-1), a co-receptor of the Nogo-receptor 1 (NgR1) complex, showed increased survival of dopaminergic neurons and functional improvements after induction of parkinsonian symptoms (Inoue et al., 2007). In line with this study, antagonizing the NgR1 significantly increased dopaminergic cell numbers and their morphological complexity in primary VM cultures (Seiler et al., 2013). Therefore, in the present work, we investigated the potential of Nogo-A neutralization on graft survival and function in a rat model with induced parkinsonian symptoms. We demonstrate here that neutralization of Nogo-A significantly promoted sprouting of grafted dopaminergic cells into the host brain and led to robust functional improvement of asymmetric forelimb use. Our results support the concept of Nogo-A inhibition as a relevant strategy for transplantation approaches in PD.

## Materials and Methods

### Animals

Female Wistar rats (Janvierlabs, France) were housed at 12 h light dark cycle with food and water *ad libitum*. For the preparation of the transplants, pregnant Wistar rats were purchased from Janvier Labs (France). All experiments were carried out in the light phase and in accordance with the guidelines of the Animal Research Ethics Committee of the Canton Berne, Switzerland, and the University of Bern Animal Care and Use Committee, Switzerland.

### Experimental Design

**Figure 1:**
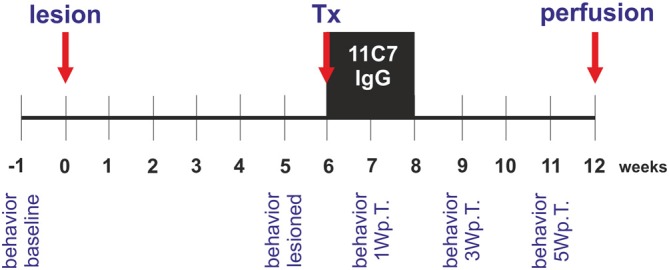
**Experimental design of the study.** Rats received a unilateral intra-striatal 6-hydroxydopamine (6-OHDA) injection (lesion) and 6 weeks later the animals were transplanted with ventral mesencephalic (VM) free-floating roller-tube cultures (Tx). Concomitantly, rats were implanted with mini-osmotic pumps releasing neutralizing Nogo-A antibodies (11C7) or control antibodies (IgG) for a period of 2 weeks (black box). Six weeks after the transplantation the rats were perfused (perfusion) and their brains removed for histological analyses. Behavior by means of the cylinder test was assessed 1 week before the lesion (behavior baseline), 5 weeks after the lesion (behavior lesioned) and 1, 3 and 5 weeks after the transplantation (behavior 1 Wp.T.; behavior 3 Wp.T.; behavior 5 Wp.T.).

To induce a unilateral reduction of dopaminergic neurons in the substantia nigra, animals received 6-hydroxydopamine (6-OHDA) injections into the right striatum. Six weeks after the lesion, each rat was grafted with half a fetal ventral mesencephalon transplant into the right striatum and received a mini-osmotic pump that injected Nogo-A neutralizing antibodies 11C7 or control antibodies (IgG; kind gifts from Dr. Anis Mir, Novartis, Switzerland) over a period of 2 weeks into the right lateral ventricle. Six weeks after the transplantation the rats were perfused and their brains used for histological analyses. Behavior (cylinder test) of the rats was assessed 1 week before (baseline) and 5 weeks after the lesion (lesioned) and 1, 3 and 5 weeks after the transplantation (Figure 1).

### Parkinson’s Disease Rat Model

Female Wistar rats (200–270 g; Janvierlabs, France) were anesthetized with Isoflurane (75% N_2_O, 20% O_2_, 4.5–5%) followed by an intraperitoneal (i.p.) injection of Narketan (75 mg/kg; Vétoquind AG, Ittigen, CH, Switzerland) and Xylaxine (5 mg/kg; Vétoquind AG, Ittigen, CH, Switzerland) and a subcutaneous (s.c.) injection of Buprenorphine (0.5 mg/kg; Reckitt Benckiser AG, Wallisellen, Switzerland) applied 30 min before surgical intervention. Thereafter, the rats were placed in a stereoscopic frame (Stoelting Co.) on a heating pad. Each rat received an injection of 4 μl of 6-OHDA (20 mM 6-OHDA; H116 Sigma-Aldrich Chemie GmbH) through a small burr hole created in the skull into the right striatum. The injection was performed over 4 min using a 10 μl Hamilton syringe. The following coordinates in relation to bregma were used (Paxinos Watson rat brain atlas): anterior 1.0 mm, lateral 3.0 mm and 5.0 mm ventral to the dura, the incisor bar was set at 0.0 mm. After the surgery rats were allowed to recover for 5 weeks.

### Preparation of Transplants

The organotypic fetal rat VM free-floating roller-tube culture technique was used for the preparation of the grafts as previously described (Andereggen et al., 2009). In brief, pregnant Wistar rats were anesthetized with Isoflurane (75% N_2_O, 20% O_2_, 4.5–5%) followed by an i.p. injection of Narketan (120 mg/kg) and Xylaxine (20 mg/kg). After removal of the fetus by cesarean section, the ventral mesencephalon was dissected out of the fetal brain with the help of a stereoscopic microscope, cut into four equally sized pieces corresponding to two rostral and two caudal portions and individually placed into gas-permeable conical plastic tubes (Falcon), supplied with 1 ml of culture medium consisting of 55% DMEM, 32.5% Hank’s balanced salt solution (HBSS; Gibco), 0.3% glucose, 10% fetal calf serum (FCS; Gibco) and 1% 0.01 M HEPES (Merck) as well as antibiotics/antimycotics (No. 061-05240 D; Gibco). Thereafter, the tubes were placed into a roller drum and grown for 7 days in an incubator at 37°C in a 5% CO_2_ atmosphere as described in detail previously (Spenger et al., 1994). The medium was changed after 2 and 5 days *in vitro*.

### Transplantation

Six weeks after the intrastriatal 6-OHDA lesions rats were anesthetized with Isoflurane (75% N_2_O, 20% O_2_, 4.5–5%) followed by an i.p. injection of Narketan (75 mg/kg) and Xylaxine (5 mg/kg). A s.c. injection of Buprenorphine (0.5 mg/kg) was given 30 min before the operations. Thereafter, the rats were mounted on a stereoscopic frame on a heating pad. Each rat received an intrastriatal graft consisting of one rostral and one caudal VM free-floating roller tube culture corresponding to half of a ventral mesencephalon from one embryo to assure roughly equal amounts of dopaminergic neurons in the grafts. The following coordinates in relation to bregma (Paxinos Watson rat brain atlas) were used: anterior 1.0 mm, lateral 2.7 mm and 4.5 mm ventral to the dura, the incisor bar was set at 0.0 mm. After the transplantation procedure, mini-osmotic pumps (2 ml2, Alzet osmotic pumps, DURECT Corporation ALZET Osmotic Pumps) were implanted under the skin and the cannulas subcutaneously connected to the skull into the right ventricle of each rat (Alzet brain infusion kit2), according to the following coordinates in relation to bregma (Paxinos Watson rat brain atlas): posterior 0.8 mm, lateral 1.6 mm and 3.5 mm ventral to the dura, the incisor bar was set at 0.0 mm. The mini-osmotic pumps were filled with either monoclonal mouse Nogo-A antibodies (11C7; 1 mg/ml) or control mouse IgG (1 mg/ml) and administered these substances continuously during the following 2 weeks (flow rate: 5 μg/h). The animals were randomly assigned to the two groups (*n* = 5 for each group) and let to recover for 1 week.

### Cylinder Test

To analyze the asymmetry in forelimb use, as observed after unilateral lesions, the cylinder test is a reliable measure for analysis of 6-OHDA induced behavioral changes in animal models of PD (Brooks and Dunnett, 2009; Cordeiro et al., 2010; Schaar et al., 2010). Behavior was assessed 1 week before the lesion (baseline), 5 weeks after the lesion (lesioned) and 1, 3 and 5 weeks after the transplantation (1 Wp.T., 3 Wp.T. and 5 Wp.T., respectively). In brief, rats were placed in a transparent cylinder (diameter 30 cm and height 41 cm) and were video recorded for 10 min. Mirrors were placed behind the cylinder to allow a 360° view on the cylinder walls. The number of wall touches with the left, the right or both paws together was counted by a researcher blinded to the treatment groups. In order to discriminate between a meaningful physiological movement and an accidental touch, only contacts in which the rat supported its body weight on the forelimb with extended digits were counted. Furthermore, rats that touched the wall less than 20 times during the 10 min period were excluded from the analysis (Schallert et al., 2000; lesioned: one animal from the IgG group with 16 touches; 1 Wp.T.: one animal from the 11C7 group with 13 touches; 3 Wp.T.: one animal from the 11C7 group with 13 touches; 5 Wp.T.: one animal from the IgG group with 14 touches and one animal from the 11C7 group with 14 touches). The percentage of left wall touches are calculated according to the formula: [(left + $\frac{1}{2}$ of both paw touches)/(left + right + both paw touches)] * 100 as previously described (Boix et al., 2015).

### Perfusions

Six weeks after the transplantation, the rats were anesthetized with Isoflurane (75% N_2_O, 20% O_2_, 4.5–5%) followed by an i.p. injection of Narketan (75 mg/kg) and Xylaxine (5 mg/kg). Just prior to opening the thorax the rats received an i.p. injection of Fentanyl (0.005mg/kg, Janssen-AG, Zug, CH, Switzerland). Thereafter, the rats were transcardinally perfused with 200 ml ice cold 0.1M phosphate buffer saline (PBS, pH 7.4) containing heparin (1000 I.E./100 ml, NOVO Nordisk) followed by 250 ml 4% paraformaldehyde in 0.1M PBS. The brains were removed from the skull and placed in 4% paraformaldehyde overnight and thereafter cryoprotected in 10% sucrose-PBS solution.

### Immunohistochemistry

The brains were cut on a cryostat (Leica CM 1900) into 30 μm thick coronal slices and mounted on Superfrost slides (Thermo Scientific) so that on one slide 3 brain slices were mounted (one 180 μm apart from the next one). Brain sections were washed 4× in PBS and blocked with 10% horse serum in 0.1% Triton-PBS. Primary and secondary antibodies were incubated in a 0.1% Triton-PBS solution containing 2.5% horse serum. After overnight incubation with the mouse monoclonal anti-tyrosine hydroxylase (TH) antibody (1:1000, Millipore) and/or the polyclonal rabbit anti-GIRK2 antibody (1:200, Alomone), the selected sections were washed 4× in PBS and incubated for 2 h with a secondary biotinylated anti-mouse IgG (1:200, Vector Laboratories) antibody. Subsequently, the slides for the TH staining only were washed in PBS, incubated in a solution of 10% methanol and 3% hydrogenperoxide in PBS to block the endogenous peroxidase and washed again in PBS. Following incubation with an avidin-biotin-complex (7 μl/ml; Vectastain ABC-Peroxidase KIT, Vector Labs) for 1 h, specifically bound antibodies were visualized with a metal-enhanced 3, 3′-diaminobenzidine substrate kit (Pierce, 34002, Life Technologies). Sections were dehydrated in alcohol, cleared in xylene and mounted in Eukitt (O. Kindler GmbH, Freiburg, Germany). The slides for the TH/GIRK2 co-localization analyses were washed 4× with PBS and incubated for 2 h with the Alexa-Fluor 594 nm donkey conjugated anti-mouse IgG (1:250, Molecular Probes) and the Alexa-Fluor 488 nm donkey conjugated anti-rabbit IgG (1:250, Molecular Probes) antibodies. Cell nuclei were counterstained with Hoechst 33342 (Invitrogen, Molecular Probes) at 1:10,000. Thereafter, the sections were washed in PBS 4 × 10 min and covered with 50% PBS-glycerol mounting media. Fluorescence pictures were taken using a Zeiss Laser scanning confocal microscope (LSM 710). Brightness, saturation, and sharpness of presented images were adjusted only as necessary to best replicate the immunostaining as viewed directly under the microscope (Gombash et al., 2012).

### Histological Analyses

All analyses were done by a researcher blinded to the treatment groups using a calibrated neuron tracing software (Cellsense Dimension, Olympus) and a microscope (Olypmus DP72) equipped with a motorized stage that was connected to a digital camera (Olympus).

#### Estimation of TH Positive Cells in the SNc

The estimation of the extent of the lesion was done as described previously by Tronci et al. (2012). In brief, brain sections of each animal were selected corresponding to the following coordinates in relation to bregma (Paxinos Watson rat brain atlas): posterior 4.8 mm (three sections analyzed, range 4.7–4.9 mm), 5.3 mm (three sections analyzed, range 5.2–5.4 mm), and 5.8 mm (three sections analyzed, range 5.7–5.9 mm). TH positive cells were counted in the SNc of the lesioned as well as the unlesioned according to stereological methods (assisted by S.A. Tschanz, Microscopy Imaging Center, Institute of Anatomy, University of Bern, Switzerland) with the help of a microscope (Olypmus DP72) equipped with a motorized stage that was connected to a digital camera (Olympus). TH positive cells with a clearly stained cell body were counted at 20× magnification excluding the cells touching the right and lower boarder of the frames. Data are expressed as percentage of TH positive cells on the lesioned side as compared to the number of TH positive neurons on the unlesioned side.

#### Histological Analysis of the Graft

Every third section containing a graft was chosen by systematic randomized sampling to determine the size of the graft, the number of TH positive fibers growing 100 μm into the host brain and the number of surviving TH positive cells in the graft. The volume of the graft was calculated as previously described (Andereggen et al., 2009). In brief, graft boundaries were traced using a calibrated neuron tracing software (Cellsense Dimension, Olympus) and after automated computation of the areas integrated to yield the graft volume. Assessment of TH positive fibers growing into the host brain was done as previously described by Andereggen et al. (2009). In brief, TH positive fibers originating in the graft were followed up to a virtual line drawn 100 μm from the graft boundary with a length of 300 μm and counted if they crossed this line. The counts were done at four sites, i.e., medial, lateral, dorsal and ventral from the border of the graft using a 10× magnification. The number of TH positive cells throughout the grafts was counted according to stereological methods (assisted by S.A. Tschanz, Microscopy Imaging Center, Institute of Anatomy, University of Bern, Switzerland) with the help of a microscope (Olypmus DP72) equipped with a motorized stage that was connected to a digital camera (Olympus). The cells were counted at 20× magnification excluding the ones touching the right and lower boarder of the frames. Only cells with a distinct immunoreactivity and a clear neuronal shape were included for analysis. The co-localization rate of TH positive neurons with GIRK2 was analyzed as described previously (Andereggen et al., 2009). In brief, the most central slides containing three brain sections of each graft were selected and stained for TH and GIRK2. The analysis was done with the help of a microscope (Olypmus DP72) equipped with a motorized stage that was connected to a digital camera (Olympus) at 20× magnification excluding the cells touching the right and lower boarder of the frames and only positive cells with a clear immunoreactivity and a clear neuronal shape were chosen for co-localization analyses. Data are expressed as percentage of total TH positive cell numbers.

#### Estimation of TH Positive Fibers in the Striatum

The density of TH positive fibers in the striatum was assessed as described previously with slight modifications (Winkler et al., 1996; Hoglinger et al., 2001). In brief, pictures of the lesioned and unlesioned dorsal striatum, 540 μm rostral before the first graft was detected, were taken from all animals with the help of a microscope (Olypmus DP72) that was connected to a digital camera (Olympus). The pictures were converted to 8 bit black and white images and inverted using the Fiji software. The mean gray intensity of the dorsal striatum of the lesioned and unlesioned side was measured in a defined area (100,000 μm^2^). To account for non-specific background staining, the mean gray value in a defined area (30,000 μm^2^) in the corpus callosum was measured. First the mean gray intensity of the corpus callosum was substracted form the respective striatal mean gray intensity values. Thereafter the values of the lesioned striatum were expressed as percentage of the unlesioned side.

#### Statistical Analysis

For statistical analysis a commercially available software package was used (GraphPad Prism 6). To compare group means of several groups repeated two-way analysis of variance (ANOVA) was used, followed by Tukey’s or Bonferroni’s multiple comparison test where appropriate. Statistical significance of two groups only, was assessed by two-tailed unpaired *t*-test and the statistical significance levels are expressed as *t*_α/υ_, where α indicates the student’s *t* values and *υ* the relative degree of freedom. Statistical significance was set at *p* < 0.05. Data are presented as mean ± SEM.

## Results

### Estimated Extent of the Lesion

**Figure 2:**
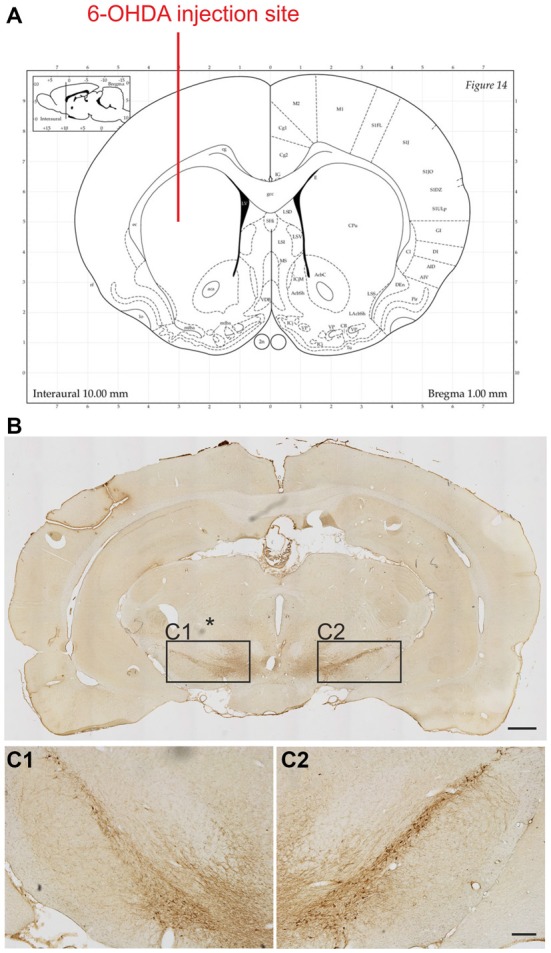
**Extent of the 6-OHDA lesion on tyrosine hydroxylase (TH) positive cell numbers in the substantia nigra.** 6-OHDA was injected into the right striatum at the following coordinates in relation to bregma (Paxinos Watson rat brain atlas): anterior 1.0 mm, lateral 3.0 mm and 5.0 mm ventral to the dura **(A)**. Representative photomicrographs showing TH positive neurons on the control and lesion (*) sides of the substantia nigra **(B)**. Boxed areas in **(B)** are shown at higher magnification **(C1,C2)**. Scale bars: 1 mm **(B)** and 200 μm **(C1,C2)**.

To determine the extent of the lesion, the surviving TH positive cells in the SNc of each rat were counted. As anticipated our lesions corresponded to an early stage of PD (Figure 2). Loss of TH positive neurons was 34.2 ± 3.0% on the lesioned side as compared to unlesioned control side with no differences observed for the three different levels analyzed in relation to bregma (34.2 ± 4.3%, 32.1 ± 4.6% and 32.9 ± 5.1%, for −4.8 mm, −5.3 mm and −5.8 mm, respectively).

### Behavioral Analysis

**Figure 3:**
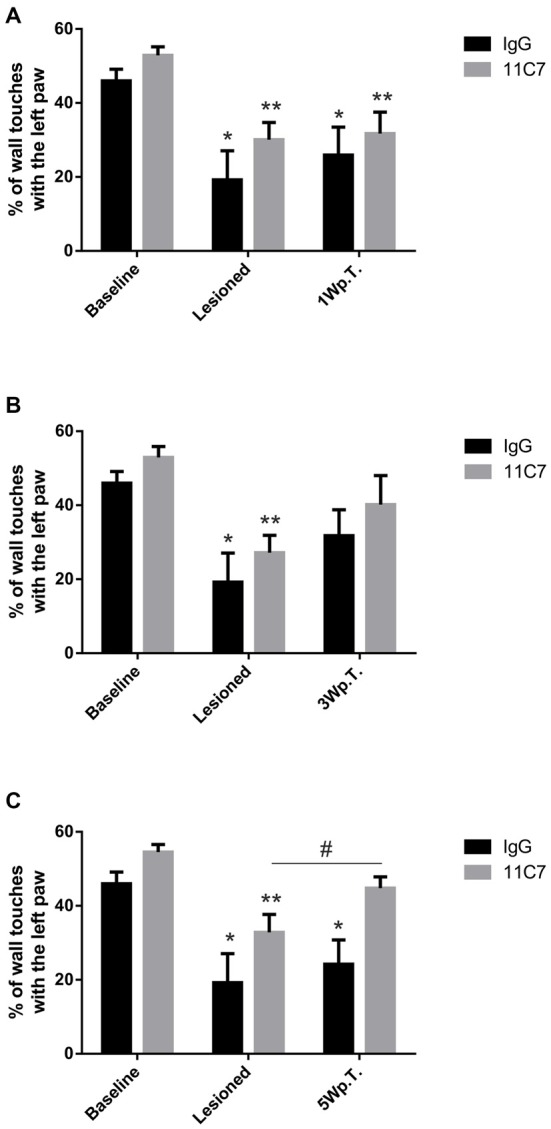
**Behavioral assessment of IgG or 11C7 treated rats by means of the cylinder test.** The outcome is shown before and after the 6-OHDA lesion as well as one (1 W.p.T.) **(A)**, three (3 W.p.T.) **(B)** and 5 weeks after the transplantation (5 W.p.T.) **(C)**. The 6-OHDA lesion resulted in significantly lower wall touches with the left paw **(A–C)**. Notably, the 11C7 treatment improved asymmetrical forelimb use 5 Wp.T as compared to after the lesion. **(C)**. In addition, 11C7 treated rats performed better than IgG treated rats 5 Wp.T. **(C)**. In contrast, IgG treated rats did not improve their behavior **(A–C)**. Data are given as mean ± SEM and expressed as percentage of corresponding baselines. *p < 0.05 vs. corresponding baselines IgG, **p < 0.05 vs. corresponding baselines 11C7, ^#^*p* < 0.05 vs. corresponding lesion 11C7.

To determine the asymmetry in forelimb use, we analyzed the rat’s behavior in the cylinder test. As expected, after the lesion the rats used the left paw (contralateral to the lesion) to touch the wall significantly less often as compared to baseline (46.0 ± 3.2 vs. 19.2 ± 7.8, % of left paw use for baseline and lesioned in the IgG group, *t*_3.2/6_ ≤ 0.05 and 52.9 ± 2.3 vs. 30.1 ± 4.7, % of left paw use for baseline and lesioned in the 11C7 group, *t*_4.4/8_ ≤ 0.01; Figure 3A). No significant difference was found between the 11C7 and the IgG groups (data not shown). One week after the transplantation no significant improvement in using the left paw in either one of the treatment groups could be observed as compared to after the lesion (19.2 ± 7.9 vs. 25.9 ± 7.5 and 30.1 ± 4.7 vs. 31.7 ± 5.8, % of left paw use for lesioned, IgG and 11C7, respectively; Time point, *F*_(2,14)_ = 25.48, *p* < 0.0001; *post hoc*, lesioned vs. IgG *p* = 0.47 and lesioned vs. 11C7 *p* = 0.94; Figure 3A). Similarly, 3 weeks after the transplantation no significant improvement in either one of the treatment groups could be detected as compared to after the lesion (19.2 ± 7.9 vs. 31.8 ± 7.0 and 27.2 ± 4.7 vs. 40.2 ± 7.9, % of left paw use for lesioned, IgG and 11C7, respectively; Time point, *F*_(2,12)_ = 21.74, *p* = 0.0001; *post hoc*, lesioned vs. IgG *p* = 0.12 and lesioned vs. 11C7 *p* = 0.09; Figure 3B). Importantly, 5 weeks after the transplantation the asymmetrical forelimb use was significantly improved in the 11C7 treated rats compared to after the lesions (19.2 ± 7.8 vs. 24.2 ± 6.5 and 32.8 ± 4.9 vs. 44.8 ± 3.0, % of left paw use for lesioned, IgG and 11C7, respectively; Time point, *F*_(2,12)_ = 29.04, *p* < 0.0001; *post hoc*, lesioned vs. IgG *p* = 0.53 and lesioned vs. 11C7 *p* = 0.05). Moreover, the 11C7 treated rats showed a trend towards more left paw uses compared to IgG treated rats (24.2 ± 6.5 vs. 44.8 ± 3.0, % of left paw use for IgG and 11C7, respectively; Treatment, *F*_(1,6)_ = 5.534, *p* = 0.057; *post hoc*, IgG vs. 11C7 *p* < 0.05; Figure 3C). In contrast, the IgG treated rats never performed significantly better during the observed test period than after the lesion and showed no recovery as compared to baseline levels (Figures 3A–C).

### Histological Analysis of the Grafts

**Figure 4:**
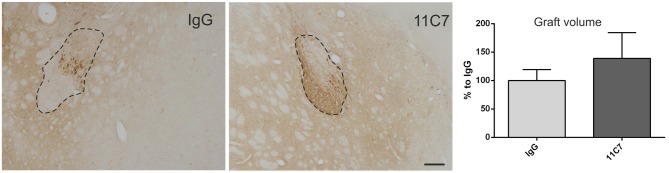
**Effects of intra-ventricular infusion of IgG or 11C7 on graft volumes.** Representative photomicrographs of grafts treated with IgG or 11C7 stained for TH. The graft boarder is highlighted with a dashed black line. Scale bar: 200 μm. No difference in graft volume was found between the two treatment groups, as demonstrated in the bar graph. Data are given as mean ± SEM and are presented as percentage of control (IgG).

**Figure 5:**
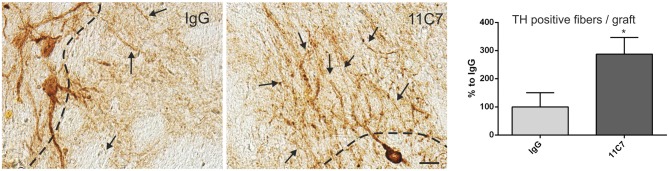
**Effects of intra-ventricular infusion of IgG or 11C7 on TH positive fiber outgrowth.** TH positive fibers from the grafts growing into the host brain (arrows) are shown on representative photomicrographs from IgG (left panel) and 11C7 (middle panel) treated animals. The black dashed line indicates the graft boarder. Scale bar: 20 μm. Note that 11C7 treatment resulted in a significantly increased number of TH positive fibers growing 100 μm into the host brain, as shown in the bar graph (right panel). Data are given as mean ± SEM and are presented as percentage of control (IgG). *p < 0.05.

**Figure 6:**
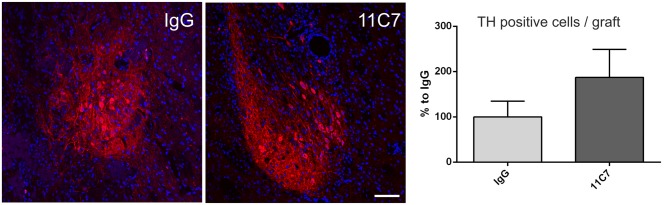
**Effects of intra-ventricular infusion of IgG or 11C7 on TH positive cell numbers in the grafts.** Representative photomicrographs showing TH positive cells (in red) in grafts from IgG and 11C7 treated animals. The sections were co-stained for the nuclear marker Hoechst (blue). Scale bar: 100 μm. Note that 11C7 treated grafts revealed a tendency towards higher numbers of TH positive cells in the graft, as shown in the bar graph. Data are given as mean ± SEM and are presented as percentage of control (IgG).

**Figure 7:**
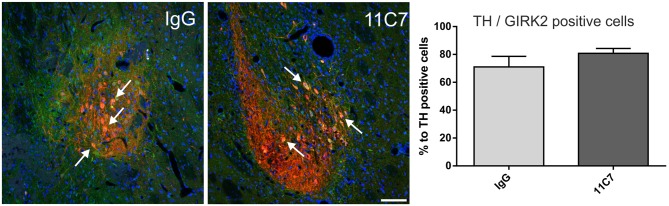
**Effects of intra-ventricular infusion of IgG or 11C7 on TH positive cells co-localizing with G-protein-coupled inward rectifier potassium channel subunit 2 (GIRK2) in the grafts.** Representative photomicrographs showing TH positive (red) and GIRK2 positive (green) cells in grafts from IgG and 11C7 treated animals. The sections were co-stained for the nuclear marker Hoechst (blue). Selected TH positive cells co-localizing with GIRK2 are highlighted with white arrows. Scale bar: 100 μm. No significant difference in number of TH positive cells co-expressing GIRK2 was observed between the two treatments, as demonstrated in the bar graph. Data are given as mean ± SEM and are presented as percentage of TH positive cells.

Both groups of treated rats showed surviving grafts that did not differ significantly in their volume (*t*_0.8/8_ = 0.448; Figure 4). Notably, 11C7 treatment significantly increased the number of TH positive fibers growing 100 μm into the host brain as compared to the IgG group (by 2.9 fold; *t*_2.4/8_ ≤ 0.05; Figure 5). Similarly, 11C7 treatment resulted in increased densities of TH positive cells in the grafts as compared to the IgG group (by 2.4 fold; *t*_1.2/8_ = 0.258) which, however, did not reach statistical significance (Figure 6). Moreover, to determine the sub-population of dopaminergic neurons that has been shown to integrate best into the host brain after transplantation, we investigated the percentage of grafted TH positive neurons co-localizing with GIRK2. The analysis revealed that about 75% of the grafted TH positive neurons co-expressed GIRK2, however, no significant differences were detected between groups (Figure 7).

### Estimation of TH Positive Fibers in the Striatum

**Figure 8:**
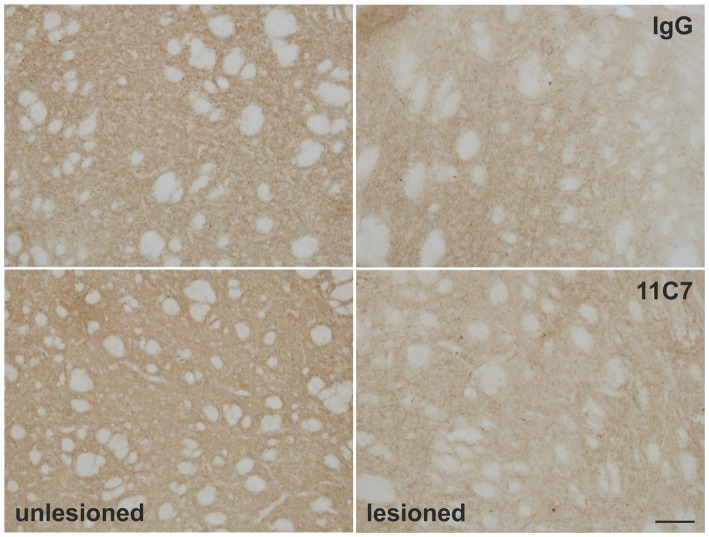
**Effects of intra-ventricular infusion of IgG or 11C7 on TH positive fibers in the dorsolateral striatum.** Representative photomicrographs showing TH positive fibers in the dorsolateral striatum on the control and lesion sides from IgG and 11C7 treated animals. Scale bar: 100 μm. No significant difference in the TH positive fiber density was observed between the two treatments.

To evaluate a potential effect of anti-Nogo-A antibody treatment on endogenous fiber growth of the surviving dopaminergic neurons, the TH positive fiber density in the dorsal striatum was analyzed. The densiometric analysis revealed, however, no difference of the mean gray intensity of TH positive fibers in the striatum of IgG and 11C7 treated rats (76.8 ± 18.9 vs. 62.7 ± 15.0, % of unlesioned side, IgG and 11C7, respectively; *t*_0.6/7_ = 0.573; Figure 8).

## Discussion

The present study shows for the first time, that transplantation of fetal VM neurons into the striatum of lesioned hemi-parkinsonian rats with concomitant neutralizing Nogo-A antibody treatment significantly increased graft derived dopaminergic fiber outgrowth and resulted in functional recovery.

In the present study, the rats were transplanted with only half a ventral mesencephalon, which corresponds to a sub-therapeutic treatment, since we have previously shown that transplantation of a whole ventral mesencephalon results in complete recovery (Meyer et al., 1998). We have chosen to transplant organotypic free-floating roller tube cultures as this method allows for effective storage of midbrain tissue, which is of importance in clinical settings where several fetuses have to be pooled to reach the amount of dopaminergic neurons needed for successful transplantations (Meyer et al., 1998; Studer, 2001). The observed lack of functional improvement after IgG treatment is thus not surprising and is consistent with our previously published data (Sautter et al., 1998; Matarredona et al., 2003). In contrast, neutralizing Nogo-A antibody treatment in a time dependent manner reversed the asymmetrical forelimb use induced by the 6-OHDA lesions over the observed period of 5 weeks. Even though we observed a roughly two fold higher survival of dopaminergic cell numbers in the graft of animals treated with neutralizing Nogo-A antibodies as compared to controls this difference did not reach statistical significance. We hence inferred that behavioral recovery was likely not due specifically to an increased survival of grafted DAergic neurons. This finding is somewhat surprising as Inoue et al. (2007) demonstrated that inhibition of LINGO-1 increases the survival of DAergic neurons in a mouse model of PD. Furthermore, blocking NgR1 or LINGO-1 was demonstrated to significantly increase the number of dopaminergic neurons in midbrain cultures (Inoue et al., 2007; Seiler et al., 2013). The difference in the outcome between our present study and the study by Inoue et al. (2007) may be due to the diverse signaling mechanisms of Nogo-A. While inhibition of LINGO-1 interferes directly with the NgR1, anti-Nogo-A antibodies target the B-20 domain of Nogo-A, thus a structure that signals independent of NgR1 (Kurowska et al., 2014). In both groups of animals, a substantial portion of surviving dopaminergic cells in the grafts co-expressed GIRK2, however, no difference was detected between groups hinting to the idea that Nogo-A neutralization did not favor a specific subpopulation of grafted neurons. Nevertheless, given the tendency for higher numbers of dopaminergic neurons in the Nogo-A group of animals one may assume that overall a larger portion of dopaminergic neurons of the GIRK2 phenotype are present in this grafts. Because graft size did not differ significantly between control and anti-Nogo-A treated rats, we suggest that the anti-Nogo-A promoted graft-derived dopaminergic fiber outgrowth contributed primarily to functional recovery. This hypothesis is supported by the observations from other studies demonstrating functional recovery in absence of higher survival of grafted dopaminergic neurons but in presence of increased fiber outgrowth and integration (Haque et al., 1996; Zhou et al., 1997; Yurek et al., 1998). Moreover, our results showing no difference of the fiber density in the striatum between both treatment groups supports the idea that mainly the fiber outgrowth of the transplanted dopaminergic neurons contributed to the improved behavior. Nevertheless, we cannot exclude that the treatment with anti-Nogo-A antibodies only accelerates the fiber outgrowth of the transplanted dopaminergic neurons. Whether at later time points the IgG treated rats would display increased densities of TH fibers sprouting from the graft remains to be investigated. Moreover, we cannot rule out the possibility that the graft itself influenced endogenous sprouting of remaining TH positive fibers, as several groups have reported particularly with adrenal gland tissue (Bankiewicz et al., 1990; Kordower et al., 1991) Promoting integration rather than the survival of grafted dopaminergic cells might be the relevant strategy to improve efficacy of transplants. Even though our study did not specifically address for adverse effects associated with the application of neutralizing Nogo-A antibodies we did not observe any obvious changes in the behavior of the animals. Given that it has been demonstrated that intra-ventricular administration of anti-Nogo-A antibodies leads to a widespread distribution of the antibodies in the brain (Wiessner et al., 2003) possible side effects of an intra-ventricular administration of anti-Nogo-A antibodies cannot completely be excluded. Nevertheless, a similar injection route, namely intrathecal administration of anti-Nogo-A antibodies in spinal cord injured patients, has been demonstrated to be feasible and safe (Zörner and Schwab, 2010).

Our findings showing augmented fiber outgrowth after Nogo-A neutralization, are in contrast to previous reports, showing unchanged neurite length and even decreased neurite numbers in dopaminergic neurons from Nogo-A knock-out animals (Kurowska et al., 2014). It is, however, important to note that the outcome of experiments employing Nogo-A knock-out animals differs from those using Nogo-A neutralization experiments. It has been reported that Nogo gene knockout results in inferior regeneration capacity as compared to treatment of animals with neutralizing agents (Teng and Tang, 2005). The authors proposed that these differences may be explained by the potential interactions of Nogo-A with other molecules, which do not take place in knock-out animals (Teng and Tang, 2005). In this respect, interactions between Nogo-A and neurotrophic factors (Raiker et al., 2010; Sepe et al., 2014) are of particular interest in view of cell transplantation approaches for PD, as glial cell line-derived neurotrophic factor, brain-derived neurotrophic factor and nerve growth factor have been described to improve graft functions and behavior recovery in animal models of PD and in PD patients (Cunningham et al., 1994; Rosenblad et al., 1996; Yurek et al., 1996, 2009; Sautter et al., 1998; Torres et al., 2005; Emborg et al., 2008; Andereggen et al., 2009; Somoza et al., 2010; Deng et al., 2013). Hence, it is tempting to speculate that Nogo-A neutralization exerts its action not only through disinhibition of neurite outgrowth but also indirectly through elevation of neurotrophic factor signaling. Such assumptions, however, have yet to be proven in the context of cell transplantation approaches for PD and remain currently speculative.

## Conclusion

Taken together, the present report demonstrates that the continuous delivery of Nogo-A neutralizing antibodies improves dopaminergic graft function likely by increasing sprouting of grafted dopaminergic neurons into the host brain. Our findings support the view that neutralization of Nogo-A in the host brain may offer a novel and therapeutically meaningful intervention for cell transplantation approaches in PD. Further studies, however, will be necessary to evaluate the clinical potential of this mode of intracerebral delivery of anti-Nogo-A antibodies to support graft survival and function.

## Ethics Statement

The experimental animal procedure was approved by the Animal Research Ethics Committee of the Canton Bern, Switzerland.

## Author Contributions

Author’s contribution to the study and manuscript preparation includes the following. Conception and design of the work was done by SS, SDS, HRW. Acquisition of data was performed by SS, SDS, HRW. Analysis and interpretation of data was carried out by SS, SDS, HRW. The draft of the article was performed by SS and critically revised by SDS, HRW. All authors read and approved the final manuscript.

## Conflict of Interest Statement

The authors declare that the research was conducted in the absence of any commercial or financial relationships that could be construed as a potential conflict of interest.

## Abbreviations

6-OHDA: 6-hydroxydopamine
CNS: central nervous system
GIRK2: G-protein-coupled inward rectifier potassium channel subunit 2
i.p.: intraperitoneal
LINGO-1: leucine rich repeat neuronal protein 1
NgR1: Nogo-receptor 1
PBS: phosphate buffered saline
PD: Parkinson’s disease
s.c.: subcutaneous
SNc: substantia nigra pars compacta
TH: tyrosine hydroxylase
VM: ventral mesencephalic.
